# Long-Term Effect of Post-traumatic Stress in Adolescence on Dendrite Development and H3K9me2/BDNF Expression in Male Rat Hippocampus and Prefrontal Cortex

**DOI:** 10.3389/fcell.2020.00682

**Published:** 2020-07-31

**Authors:** Mingyue Zhao, Wei Wang, Zhijun Jiang, Zemeng Zhu, Dexiang Liu, Fang Pan

**Affiliations:** Department of Medical Psychology and Medical Ethics, School of Basic Medicine Sciences, Cheeloo College of Medicine, Shandong University, Jinan, China

**Keywords:** PTSD, BDNF, H3K9me2, neurodevelopment, methylation, epigenetic

## Abstract

Exposure to a harsh environment in early life increases in the risk of post-traumatic stress disorder (PTSD) of an individual. Brain derived neurotrophic factor (BDNF) plays an important role in neurodevelopment in developmental stages. Both chronic and traumatic stresses induce a decrease in the level of BDNF and reduce neural plasticity, which is linked to the pathogenesis of PTSD. Also, studies have shown that stress alters the epigenetic marker H3K9me2, which can bind to the promoter region of the *Bdnf* gene and reduce BDNF protein level. However, the long-term effects of traumatic stress during adolescence on H3K9me2, BDNF expression and dendrite development are not well-known. The present study established a model of PTSD in adolescent rats using an inescapable foot shock (IFS) procedure. Anxiety-like behaviors, social interaction behavior and memory function were assessed by the open field test, elevated plus maze test, three-chamber sociability test and Morris water maze test. In addition, neuronal development and H3K9me2/BDNF expression in hippocampus (HIP) and prefrontal cortex (PFC) were evaluated by Golgi staining, western blotting, qRT-PCR analysis and CHIP-qPCR analysis. Additionally, the Unc0642, a small molecule inhibitor of histone methyltransferase (EHMT2) was used for intervention. The results showed that the IFS procedure induced the PTSD-like behaviors in rats, resulted in fewer dendrite branches and shorter dendrite length in CA1 of HIP and PFC, increased H3K9me2 level and decreased BDNF expression in HIP and PFC. Also, although all the changes can persist to adulthood, Unc0642 administration relieved most of alterations. Our study suggests that traumatic stress in adolescence leads to immediate and long-term mental disorders, neuronal morphological changes, lower BDNF level and increased H3K9me2 level in the HIP and PFC, indicating that H3K9me2/BDNF dysfunction plays a key role in pathogenesis of PTSD.

## Introduction

Exposure to a harsh environment has a negative impact on brain structure that may lead to impairment of cognitive and emotional function in humans (Wang R. et al., 2018; Xu et al., 2019). Also, experiencing stress in early childhood may easily affect neuronal structure and function, thereby leading to substance abuse, anxiety, depression and even learning function and social communication impairments (Salzmann-Erikson and Hicdurmaz, 2017; Cowan et al., 2018; Postel et al., 2019; Ohta et al., 2020). In general, serious and life threatening injury events induce post-traumatic stress disorder (PTSD) which leads to long-term mental health problems, such as anxiety, depression and suicide (Friedman et al., 2011). The clinical symptoms of PTSD include recurrent and intrusive traumatic memories, avoidance of traumatic event-related stimuli, cognitive impairment, negative emotions, hyper-aroused state, hyper-vigilance, clinical distress, and social impairment (Richter-Levin et al., 2019). The pathogenesis of PTSD can involve many aspects, such as genetic factors, neurological, neuroendocrine and environmental factors (Pitman et al., 2012). However, dysregulation of dendrite development and synaptogenesis has been confirmed as a key factor in the etiology of PTSD (Heun-Johnson and Levitt, 2018).

Brain derived neurotrophic factor (BDNF) is a protein synthesized in the brain and widely distributed in the central and peripheral nervous systems. BDNF is considered to play an important role in neuronal differentiation, growth and development. In addition, BDNF has biological effects, such as preventing neuronal damage and death, improving neuronal pathological state, and promoting neuron regeneration (Lima Giacobbo et al., 2019; Voisey et al., 2019; Wen et al., 2020). An earlier study has shown that reduced expression levels of BDNF affect neuronal development and synaptic plasticity (Wang et al., 2015). In particularly, traumatic stress can significantly reduce the expression of BNDF in hippocampus (HIP) (Shafia et al., 2017; Alzoubi et al., 2019), impair synaptic plasticity in the prefrontal cortex (PFC) which are associated with susceptibility to trauma-related symptoms (Lguensat et al., 2019; Li et al., 2019; Zhang et al., 2019).

Many studies have shown that the expression of BDNF is involved in neuronal development and the epigenetic marker of H3K9me2/3 has a negative regulatory effect on transcription level of *Bdnf* gene (Walker et al., 2013; Kyzar et al., 2017). Recent studies have shown that H3K9me2 and its degree of methylation participate in regulating the occurrence of depression and anxiety symptoms in early maternally-separated separation young mice (Xu et al., 2018). More importantly, some studies suggest that stress-induced H3K9me2/3 undergoes irreversible changes, which have a regulatory effect on the expression of the *Bdnf* gene (Wu et al., 2019; Li et al., 2020). For example, continuous chronic exposure to morphine can trigger the methylation modification of H3K27 in the ventral segmental nucleus, which affects the transcriptional downregulation of *Bdnf* promoter sequences (Koo et al., 2015). Also, social isolation and chronic unpredictable stress induce the acetylation of H3K9 and H4K12 in their corresponding brain regions, which can reduce the expression level of BDNF and impair memory function in rats (Viana Borges et al., 2019). Such studies indicate that stress alters H3K9me2/3/BDNF signaling, which is involved in affective disorder and memory impairment. However, the role of traumatic stress in adolescence on dendrite development and H3K9me2/BDNF expression has not been elucidated. This study established a traumatic stress model in adolescent rats. In addition, Unc0642, a small molecule inhibitor of histone methyltransferase (EHMT2), was used to reduce the protein expression level of H3K9me2 during the stress procedure (Xiong et al., 2017; Wang et al., 2019). We hypothesized that traumatic stress in adolescence might induce behavioral dysfunction, dysregulation of neuronal development and maturation and changes in H3K9me2/3/BDNF expression in HIP and PFC, and those effects might persist to adulthood.

## Materials and Methods

### Animals and Drug Treatment

A total of 72, 21-day old male Wistar rats weighing 60–80 g were purchased from the Animal Center of Shandong University (Jinan, Shandong, China). Rats (5 per cage) were housed in the Animal Center, in a controlled environment (12 h day/night cycle, 23 ± 2°C) and with *ad libitum* access to food and water. The experimental procedures conformed to the guidelines of the Animal Ethics Committee of Shandong University. Unc0642, a small molecule inhibitor of EHMT2 (G9a), can reduce the protein expression level of H3K9me2. Un0642 was dissolved in dimethyl sulfoxide (DMSO), polyethylene glycol (PEG) 300 and double-distilled water (DDW). The injection (i.p) (2.5 mg/kg/d) was administered 30 min after each shock (Berkel et al., 2019; Griñán-Ferré et al., 2019; Wang et al., 2019).

### Experimental Design

**FIGURE 1:**
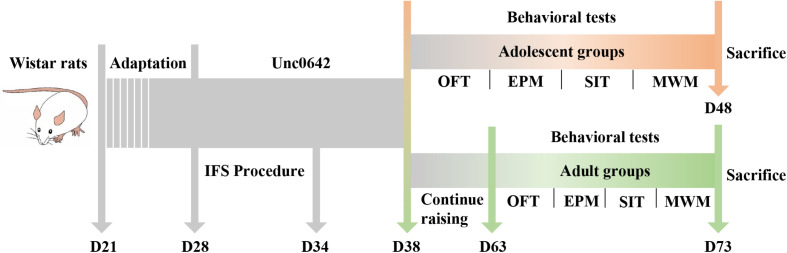
Experimental flow chart.

After 7 days of adaptive feeding, the 72 male Wistar rats were randomly divided into three groups (24/group) according to their body weight, namely the control group, PTSD group and PTSD+Unc0642 group. Rats in the PTSD group received inescapable foot shock (IFS) for 6 days, while rats in the PTSD+Unc0642 group received both IFS and an intraperitoneal injection of Unc0642, for 6 and 10 days, respectively. Rats in control group and PTSD group were received intraperitoneal injection of saline for 10 days for balancing the operation error. After modeling, rats in each of the three groups were randomly divided into adolescent groups and adult groups. Then, rats in the adolescent groups were subjected to behavioral tests. For the adolescent rat groups, rats were sacrificed on the next day after the behavioral tests and brain tissues were collected for subsequent analysis. The rats in the adult groups were raised to 63 days of age and then subjected to the behavioral tests (see the Figure 1). Therefore, there are 6 groups in this study, namely the adolescent control group (AdoC, *n* = 12), the adolescent PTSD group (AdoP, *n* = 12), the adolescent Unc0642 medicated group (AdoP+U, *n* = 12), the adult control group (AduC, *n* = 12), the adult PTSD group (AduP, *n* = 12), and the adult Unc0642 medicated group (AduP+U, *n* = 12).

### The Inescapable Foot Shock (IFS) Procedure

The IFS procedure was used to establish a PTSD animal model (Ju et al., 2017). Rats were placed in an electric shock closed box, and the feet of the rats were subjected to consecutive unavoidable shocks for 18 times in a semi-random manner. Shock time interval was random, and the interval time was 30–120 s. IFS was performed twice a day, the interval was not less than 4 h. As a precaution to eliminate error interference, the rats in the control group were treated in a discharge box only protected from light but without electric shock.

### Behavioral Tests

The exploration of new environment was evaluated by the open field test (OFT), the anxiety-like behavior was assessed by the elevated plus maze (EPM) test, the ability to explore novel environment and strange rats was evaluated using the social interaction test (SIT), and the spatial exploration and spatial memory capabilities were tested by the Morris water maze (MWM) test.

#### The Open Field Test

The open field experiment was performed to determine the autonomous movement and behavioral exploration ability of rats in a novel environment (Prut and Belzung, 2003). The rats were placed in the center of the experimental box, then the number of times of crossing, time spent in the central squares and the number of rearings of the rats were recorded for 5 min using the Smart software (SMART 2.5, Panlab). The test box was wiped with alcohol between tests in order to eliminate any residual olfactory cues left by the previously tested rat.

#### The Elevated Plus Maze Test

The elevated plus maze experiment was conducted to assess anxiety in rats (Walf and Frye, 2007). The elevated plus maze has two open arms and two closed arms that above 50 cm from the ground. The rats were placed at an elevated cross with their heads facing the open arms, and indexes of the number of times the rat entered into the open and closed arms and the time the rat in the open and closed arms in 5 min were recorded using the Smart software.

#### Three Chamber Sociability Test

Three social experiment chambers were used to test the ability to explore a novel environment and strange rats (social interaction behaviors) (Kaidanovich-Beilin et al., 2011). The experimental rectangular chamber with a top length of 120 cm, width of 60 cm, and height of 40 cm was divided into three parts of equal volume by a partition. Each partition has a small door of 20 × 10 cm. Rats were allowed to freely enter the three sections (left, center, right). Each of the left and right parts had a cage holding a strange rat. Before the experiment, rats were allowed to adapt to the experimental environment. The social experiment consisted of three phases, namely, the adaptation phase, the first phase and the second phase. In the 5 min adaptation phase, the rats were placed in the central part of the social box and the two doors were open, so that the rats could move freely to explore the social box. The first 5 min stage, the strange rat 1 is placed in either the left or right imprisonment cage, and the experimental rat is still placed in the central part of the social chamber with the two doors opened, so that the rat could freely shuttle through the two doors to explore the social chamber. The imprisonment cages that have been contacted during the adaptation phase, therefore, the number of times and time of exploring strange rat 1 represent the ability of experimental rats to explore novelty. In the second 5 min stage, the strange rat 2 is placed in the cage opposite to where the strange rat 1 was placed, and the experimental rat is still placed in the central part of the social chamber with the two doors opened, so that the rats can freely shuttle through the two doors to explore the social chamber. Compared to the strange rat 1 (the familiar rat at this stage) that has been contacted in the first 5 min stage, exploring the strange rat 2 showed the ability to explore novelty. The number of times and time that the experimental rat shuttled the left and right door are recorded, as well as the number of times and time of contact with the empty cage and the strange rat 1 and 2 in the first and second stages.

#### The Morris Water Maze Test

The Morris water maze experiment was carried out to assess spatial memory and memory function in rats (Vorhees and Williams, 2006). The MWM is a cylinder with a radius of 60 cm divided into four quadrants. The platform belongs to the fourth quadrant and is hidden 1 cm underwater. The training took place during the first 5 days. The sixth day was the experimental day. During the training period, the searching time for the platform was 1 min, if the rat had not yet found the platform in 1 min, it was artificially dragged to the platform and stay for 30 s. During the experimental period, the platform was withdrawn, and the probe trial, the number of times and time of crossing the 5th quadrant were recorded.

### Biochemical Analysis

#### Golgi Staining

After sacrificing the rats, the brains of 3 rats in each group were removed and placed immediately in 4% paraformaldehyde fixative (Servicebio, G1101) for more than 24 h and subsequently stained by Golgi staining (Golgi staining kit, Servicebio, G1069) and then photographed for analysis using the Image J 6.0 software [National Institute of Health (NIH), Bethesda, MD, United States] (Theer et al., 2014; Louth et al., 2017). Sholl analysis was used to analyze the dendrite length and branches. The CA1, CA2/3, and DG in HIP and PFC were analyzed. Briefly, the cell body of the neuron was used as the center in the 200× field of view. Then, concentric circles were made separated by a distance of 10 μm, and the sum of the intersection points of the dendrites and concentric circles (data of 10 concentric circles outside the cell) was counted. The total number of intersection points counted was considered to reflect the dendrites and the density of dendritic spines on the base dendrite in the field of 1000× was observed. Due to the uneven distribution of dendritic spines on the dendrites at all levels, observation was started from the first branch of the dendrite to the cell body, and the range of 30–90 μm was used for calculation. The number of internal dendritic spines is the density of dendritic spines per 10 μm.

#### Western Blot Analysis

Samples of 30 mg of PFC and hippocampal tissues of 4 rats from each group were placed in 1.5 mL EP tubes. Then, for each 20 mg of tissue 200–250 μL of RIPA lysis buffer (Beyotime, Shanghai, China), containing 1% of the protease inhibitor phenylmethylsulfonyl fluoride (PMSF), was added for tissue lysis. Subsequently, 2 high-pressure small steel balls were added to each EP tube, which were then placed a pre-cooled mill. The parameters used for grinding were 60 Hz and a grinding time of 60 s. After grinding to obtain a homogeneous mixture, the lysate was centrifuged at 12,000 rpm in a cold centrifuge at 4°C for 25 min. Afterward, the supernatant was carefully removed and aliquoted into 80 μL per tube. Protein concentration was determined by the bicinchoninic acid assay (BCA) method. Before loading the lysate samples onto the sodium dodecyl sulfate-polyacrylamide gel electrophoresis (SDS-PAGE) gel for protein separation, each lysate sample containing the same amount of protein was mixed with 5 × loading buffer and boiled for 10 min. Then, the proteins in each sample were separated on 8 and 10% SDS-PAGE gels and subsequently transferred onto a 0.22 or 0.45 μm polyvinylidene fluoride (PVDF) membrane. Then, after blocking the membrane with 5% skim milk for 2 h, the primary antibody against BDNF (CY5577, Abways Biotechnology Co., Ltd., Shanghai, China) or against H3K9me2 (ab1220, Abcam, Cambridge, United Kingdom) was added and the membrane was incubated overnight at 4°C (Berkel et al., 2019). Subsequently, after washing the membrane three times with tris-buffered saline Tween 20 (TBST) for 10 min each time, the secondary antibody was added, and incubated for 1 h at room temperature. The analysis of the protein bands grayscale values was performed by Image J software.

#### RNA Extraction and qRT-PCR

The brain tissues of 2 rats and a half of brain tissue of 1 rat from each group were used to test qRT-PCR. Total RNA was extracted from 20 mg of PFC and hippocampal tissue samples. Each tissue sample was placed in a 2 mL EP tubes containing 500 μL of Trizol (RR003, HONBIOTECH, Jinan, China) and two small steel balls. The tissue in each EP tube was ground using a pre-cooled grinder, at 60 Hz for 60 s, to obtain a homogeneous mixture. Then, 200 μL of chloroform were added to each homogenate mixture, followed by vigorous shaking, and incubation at room temperature for 5 min to fully extract RNA. Afterward, the tubes were centrifuged in a cold centrifuge and the supernatant was transferred into a new centrifuge tube, and mixed with an equal volume of isopropyl. At this step, the samples can be store at −80°C or the RNA extraction can be completed by centrifuging, discarding the supernatant, and washing the pellet with 75% ethanol (prepared in DEPC water) to obtain the RNA. The RNA concentration and integrity were determined using a spectrophotometer. The total RNA was reverse transcribed into cDNA using a reverse transcription kit (ReverTra Ace^®^ qPCR RT Master Mix with gDNA Remover, FSQ-301, TOYOBO, Japan). The prepared cDNA was used to measure gene expression by qRT-PCR analysis using a Bio-Rad qRT-PCR system (Bio-Rad Laboratories, Hercules, CA, United States). The qRT-PCR generated data were used to determine the relative expression of the genes of interest using the 2^–ΔΔ^*^*C*^*^T^ method (Livak and Schmittgen, 2001). The sequence of primers used is as follows: Rat-Bdnf-F 5′-GTCCCGGTATCAAAAGGCCA-3′; Rat-Bdnf-R 5′-ATCCTTATGAACCGCCAGCC-3′. Rat-β-actin-F 5′-CTCTGTGTGGATTGGTGGCT-3′, Rat-β-actin-R 5′-CGCAGCTCAGTAACAGTCCG-3′.

#### CHIP-qPCR

**FIGURE 2:**
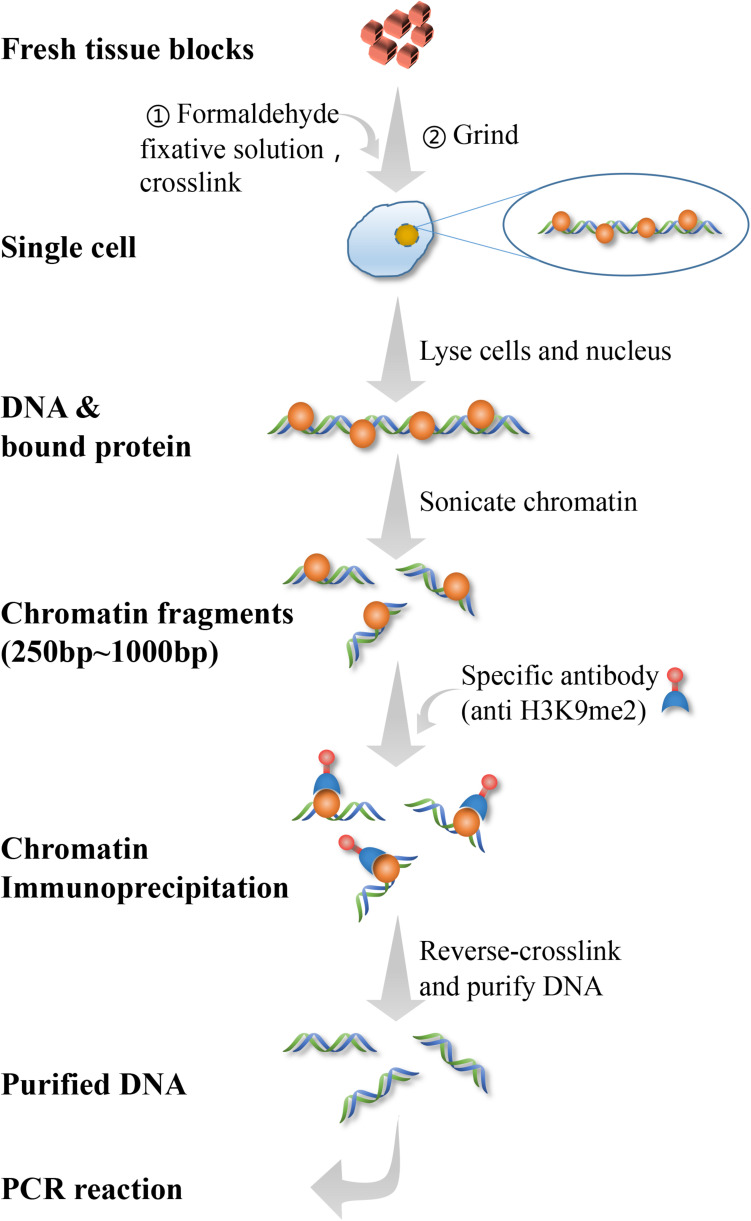
CHIP-qPCR flow chart.

The PFC and hippocampal tissues of 2 rats and a half of brain tissue of 1 rat from each group were used to test CHIP-qPCR. Chromatin co-precipitation is a protein localization technique that analyzes binding to specific regions within the genome. Chromatin components can be selectively enriched using antibodies specific to the protein of interest. After enriching the protein of interest, pull-down technology is used to isolate the protein-interacting DNA regions. Then, qPCR is performed on the pull-down DNA to determine that the protein of interest is bound to the gene and its binding site is determined. According to CHIP kit (56383, Cell Signaling Technology, Inc., United states), this experiment was performed within 1 week sacrificing the rats. A total of 60–75 mg of tissue was manually cut into 1–2 mm pieces of tissue. Then, after adding CHIP cell lysis buffer, each tissue sample was manually ground into a single cell suspension. Afterward, ultrasonic lysis was performed after adding CHIP nuclear lysis buffer to obtain the ideal chromatin fragment of 150–1,000 bp. Then the immunoprecipitation reaction was performed, followed by de-crosslinking after overnight treatment with magnetic beads and antibodies. A DNA purification kit was used to obtain purified DNA to be used for CHIP-qPCR analysis and determine the enrichment rate. This technology is used to further demonstrate the relationship between H3K9me2 and the *Bdnf* gene promoter, in order to determine whether H3K9me2 directly regulates the expression of the *Bdnf* gene and BDNF protein (Tsankova et al., 2004; Cowan et al., 2018). The experimental procedure of CHIP was showed in Figure 2. The sequence of primers used as follows: Bdnf-PF 5′-TGATCATCACTCACGACCACG-3′; Bdnf-PR 5′-CAGCCTCTCTGAGCCAGTTACG-3′.

#### Statistical Analysis

Quantitative data are expressed as the mean ± SEM (standard error of the mean). One-way analysis of variance (ANOVA) followed by Tukey’s *post hoc* test was used for statistical analysis, except for the average escape latency of the MWM test. For the MWM test, the average escape latency in the 5 training days was used for three-way repeated-measures ANOVA. Differences were considered significant when the *P* value was < 0.05.

## Results

### Behavioral Tests

#### Traumatic Stress Procedure and Effect of the Unc0642 on Exploratory and Anxiety-Like Behaviors Both in Adolescent and Adult Rats

**FIGURE 3:**
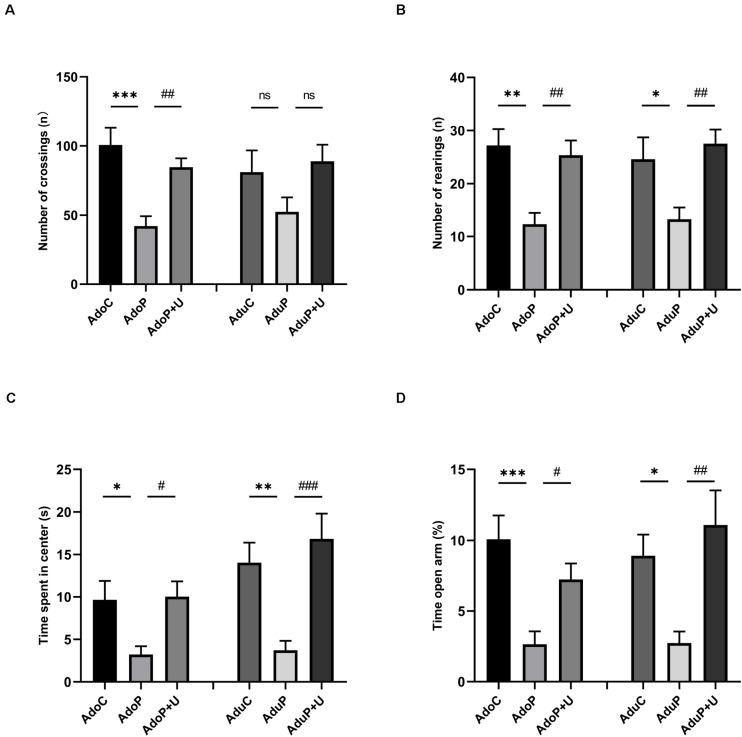
Traumatic stress procedure and Unc0642 administration effect on depressive-like and anxiety-like behaviors both in adolescent and adult rats. **(A)** The number of crossings in OFT. **(B)** The number of rearings in OFT. **(C)** Time spent in center in OFT. **(D)** Time in open arm (%) in EPM test. Results were presented as mean ± SEM (*n* = 12 each group). *p < 0.05, **p < 0.01, ***p < 0.001, and ^#^*p <* 0.05, ^##^*p <* 0.01, ^###^*p <* 0.001; ns, none significant (Tukey’s test).

The results of the OFT are shown in Figure 3. Traumatic stress reduced the number of crossings in the adolescent rats [*F*(2, 33) = 11.20, *P* < 0.05] (*post hoc*, *P* = 0.002) compared with the adolescent controls, and Unc0642 administration attenuated this behavioral change (*post hoc*, *P* = 0.006). However, there has no change in the number of crossings for adult rats [*F*(2, 33) = 2.224, *P* > 0.05] (*post hoc*, *P* = 0.2726) (Figure 3A). In addition, traumatic stress reduced the number of rearings in adolescent rats [*F*(2, 33) = 9.103, *P* < 0.05] (*post hoc*, *P* = 0.0012) and Unc0642 administration mitigated this behavior in adolescent rats (post hoc, *P* = 0.0046) compared with stressed rats. Meanwhile, traumatic stress decreased the number of rearings in the adult group [*F*(2, 33) = 5.90, *P* < 0.05] (*post hoc*, *P* = 0.0037) and Unc0642 treatment increased the number of rearings in this group (*post hoc*, *P* = 0.0073) (Figure 3B). Also, traumatic stress reduced the time spent in the center in adolescent rats [*F*(2, 33) = 4.683, *P* < 0.05] (*post hoc*, *P* = 0.0382) and Unc0642 treatment alleviated this behavioral alteration in adolescent rats (*post hoc*, *P* = 0.0271). Additionally, traumatic stress decreased the time spent in the center in adult rats [*F*(2, 33) = 9.27, *P* < 0.05] (*post hoc*, *P* = 0.0079) compared with control rats and Unc0642 treatment attenuated this index (*post hoc*, *P* = 0.0008) (Figure 3C).

The results of the EPM test are shown in Figure 3D. Traumatic stress decreased the time in the open arm (%) in adolescent rats [*F*(2, 33) = 8.59, *P* < 0.05] (*post hoc*, *P* = 0.0007) and Unc0642 treatment with mitigated this behavior in adolescent rats (*post hoc*, *P* = 0.0414). Traumatic stress also decreased the time in the open arm (%) in adult rats [*F*(2, 33) = 6.37, *P* < 0.05] (*post hoc*, *P* = 0.0407) and Unc0642 treatment relieved this behavior in adult rats (*post hoc*, *P* = 0.0044) (Figure 3D).

#### Traumatic Stress Procedure and Effect of Unc0642 on Social Interaction Behaviors Both in Adolescent and Adult Rats

**FIGURE 4:**
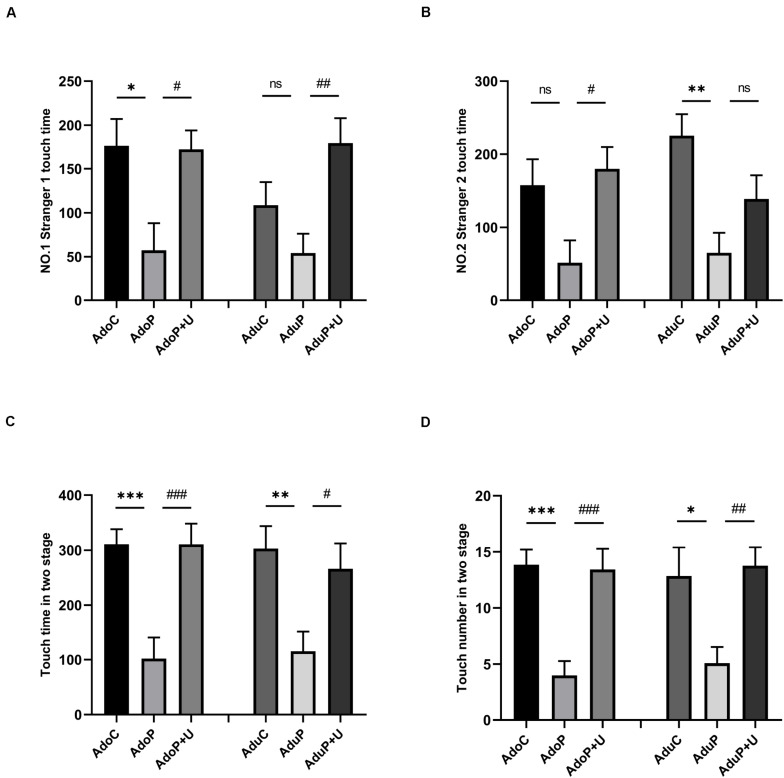
Traumatic stress procedure and Unc0642 administration effect on social interaction changes both in adolescent and adult rats. **(A)** Stranger 1 touch time in first stage (NO.1) in three-chamber sociability test. **(B)** Stranger 2 touch time in second stage (NO.2) in three-chamber sociability test. **(C)** Touch time in two stages. **(D)** Touch number in two stages. Results were presented as mean ± SEM (*n* = 12 each group). *p < 0.05, **p < 0.01, ***p < 0.001 and ^#^*p <* 0.05, ^##^*p <* 0.01, ^###^*p <* 0.001; ns, none significant (Tukey’s test).

The results of the SIT are shown in Figure 4. Traumatic stress decreased the touching time in first stage in adolescent rats [*F*(2, 33) = 5.75, *P* < 0.05] (*post hoc*, *P* = 0.0142) and treatment with Unc0642 alleviated this behavior (*post hoc*, *P* = 0.0182) compared with adolescent stressed rats. Meanwhile, in adult groups, the effect of traumatic stress on the decrease of the touching time showed no significant statistical differences [*F*(2, 33) = 5.877, *P* > 0.05] (*post hoc*, *P* = 0.3089), but treatment with Unc0642 relieved the behavior in adult rats (*post hoc*, *P* = 0.0047) (Figure 4A).

In addition, traumatic stress decreased the touching time in second stage in adolescent rats, but the differences were not statistically significant [*F*(2, 33) = 4.58, *P* > 0.05] (*post hoc*, *P* = 0.0640), and Unc0642 administration mitigated this behavior in adolescent rats (*post hoc*, *P* = 0.0209) compared with stressed rats. Also, in adult groups, traumatic stress decreased the touching time in second stage in adult rats [*F*(2, 33) = 7.151, *P* < 0.05] (*post hoc*, *P* = 0.0018), but treatment with Unc0642 had no effect on relieving this behavior in adult rats (*post hoc*, *P* = 0.2068) (Figure 4B).

Traumatic stress also decreased the total touching time in first and second stages in adolescent rats [*F*(2, 33) = 11.94, *P* < 0.05] (*post hoc*, *P* = 0.0005) and Unc0642 administration attenuated this behavior in adolescent rats (*post hoc*, *P* = 0.0005) compared with stressed rats. In adult groups, traumatic stress decreased the total touching time in first and second stages [*F*(2, 33) = 5.62, *P* < 0.05] (*post hoc*, *P* = 0.0088) and treatment with Unc0642 alleviated this behavior in adult rats (*post hoc*, *P* = 0.0404) (Figure 4C).

Additionally, traumatic stress reduced the total touching number in first and second stages in adolescent rats [*F*(2, 33) = 13.45, *P* < 0.05] (*post hoc*, *P* = 0.0002) and treatment with Unc0642 attenuated this behavior in adolescent rats (*post hoc*, *P* = 0.0003) compared with stressed rats. In adult groups, traumatic stress reduced the total touching number in first and second stages [*F*(2, 33) = 5.98, *P* < 0.05] (*post hoc*, *P* = 0.0217) and Unc0642 administration relieved the behavior in adult rats (*post hoc*, *P* = 0.0095) (Figure 4D).

#### Traumatic Stress Procedure and Effect of Unc0642 on Spatial Learning and Memory Both in Adolescent and Adult Rats

**FIGURE 5:**
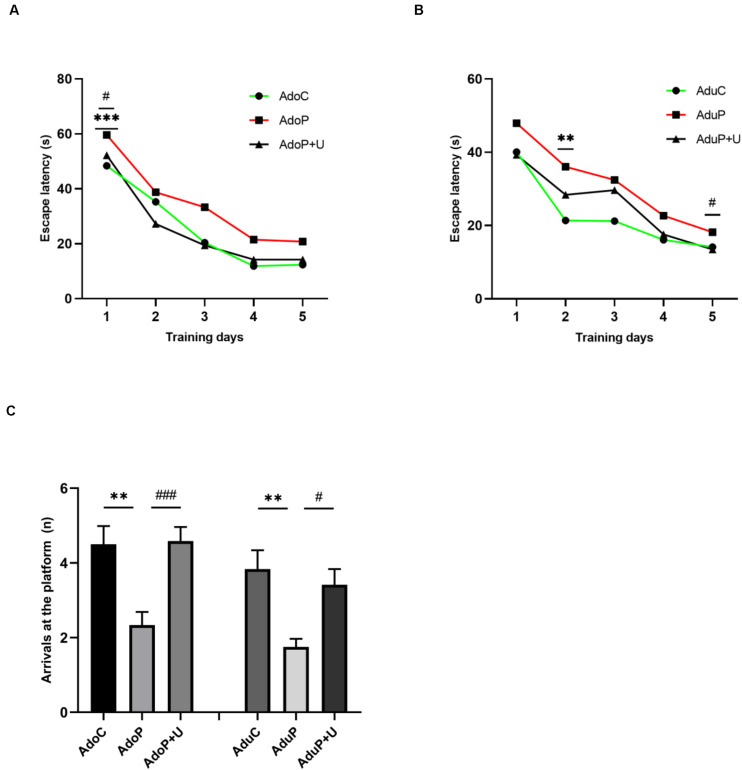
Traumatic stress procedure and effect of Unc0642 on spatial learning and memory impairment both in adolescent and adult rats. **(A)** Escape latency in MWM test in adolescent rats. **(B)** Escape latency in MWM test in adult rats. **(C)** The number of arrivals at the platform in MWM test. Results were presented as mean ± SEM (*n* = 12 each group). **p < 0.01, ***p < 0.001 and ^#^*p <* 0.05, ^###^*p <* 0.001 (Tukey’s test).

The results of the MWM test are shown in the Figure 5. In adolescent groups, escape latency of the three groups in the training period was shortened day by day [*F*(4, 132) = 78.22, *P* < 0.0001], and there was no relation between time and stress [*F*(8, 132) = 1.068, *P* > 0.05]. Traumatic stress increased the time of the average escape latency on the first day (*P* < 0.001) and treatment with Unc0642 alleviated this behavior on the first day (*post hoc*, *P* = 0.0350) compared with stressed rats. Additionally, there were differences between these groups [*F*(2, 33) = 7.50, *P* < 0.05] (Figure 5A). In adult groups, the escape latency of the three groups in the training period was shortened day by day [*F*(4, 132) = 37.47, *P* < 0.0001], and there was no relation between time and stress [*F*(8, 132) = 0.97, *P* > 0.05]. In addition, traumatic stress increased the time of the average escape latency on the second day (*post hoc*, *P* = 0.0020) and Unc0642 administration not relieved this behavior on the second day (*post hoc*, *P* = 0.1150) compared with stressed rats, but Unc0642 administration relieved this behavior on the fifth day (*post hoc*, *P* = 0.0460). Additionally, there were differences between these groups [*F*(2, 33) = 19.36, *P* < 0.0001] (Figure 5B). Traumatic stress reduced the number of arrivals at the platform in adolescent rats [*F*(2, 33) = 9.68, *P* < 0.05] (*post hoc*, *P* = 0.0020) and treatment with Unc0642 mitigated this behavior in adolescent rats (*post hoc*, *P* = 0.0013) compared with stressed rats. Also, in adult groups, traumatic stress reduced the number of arrivals at the platform [*F*(2, 33) = 7.66, *P* < 0.05] (*post hoc*, *P* = 0.0022) compared with control rats and Unc0642 administration attenuated this behavior in adult rats (*post hoc*, *P* = 0.0153) (Figure 5C).

### Biochemical Determination

#### Traumatic Stress Procedure and Effect of Unc0642 on Neuronal Morphological Changes of Golgi Staining Both in Adolescent and Adult Rats

**FIGURE 6:**
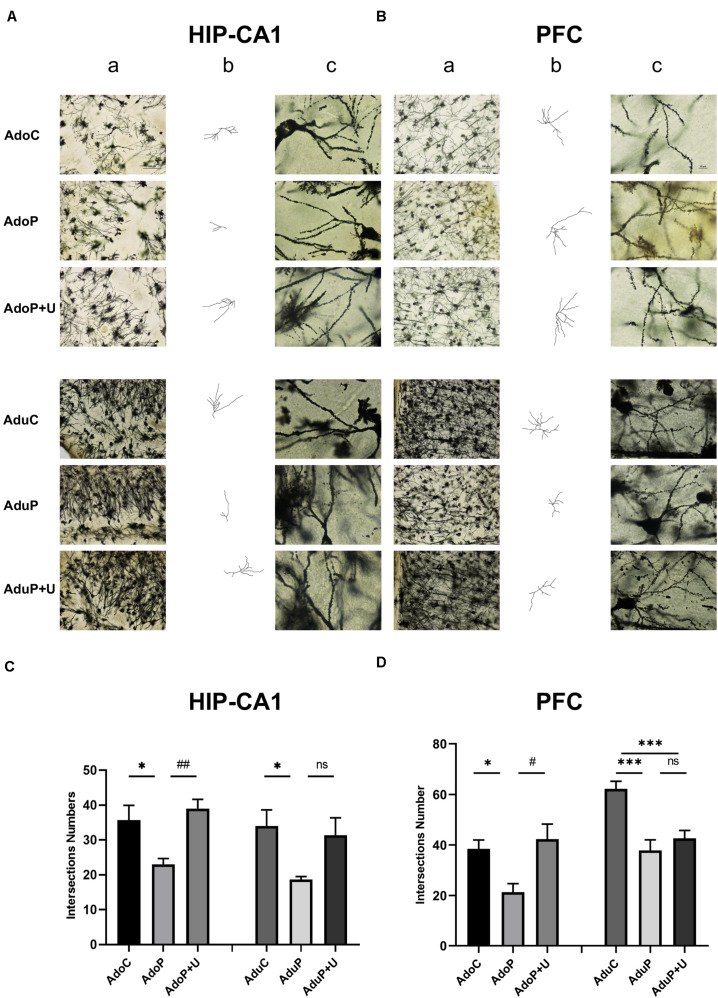
Traumatic stress procedure and effect of Unc0642 on neuronal morphological changes of Golgi dyeing both in adolescent and adult rats. **(A)** Golgi dyeing in hippocampus-CA1 of adolescent and adult rats. **(B)** Golgi dyeing in prefrontal cortex of adolescent and adult rats. **(C)** Intersections number of adolescent and adult rats. **(D)** Intersections number of adolescent and adult rats. a. 200× in Golgi dyeing; b. Black and white picture; c. 1000× in Golgi dyeing. Results were presented as mean ± SEM (*n* = 3 each group). *p < 0.05, ***p < 0.001 and ^#^*p <* 0.05, ^##^*p <* 0.01; ns, none significant (Tukey’s test).

The total number of intersections represent the length of each neuron and its degree of branch richness, which indicate the neuronal development. The longer length and the more branches reveal its better development. The results are shown in Figure 6 (HIP-CA1) and Supplementary Figure 1 (HIP-CA2/3 and DG), traumatic stress reduced the total number of intersections of the HIP-CA1 in adolescent rats [*F*(2, 15) = 7.62, *P* < 0.05] (*post hoc*, *P* = 0.0260) and treatment with Unc0642 relieved these changes in adolescent rats (*post hoc*, *P* = 0.0057) compared with stressed rats. In adult groups, traumatic stress reduced the number of intersections of the HIP-CA1 [*F*(2, 15) = 4.21, *P* < 0.05] (*post hoc*, *P* = 0.0399), but treatment with Unc0642 had no effect on relieving these changes in adult rats (*post hoc*, *P* = 0.0959) compared with stressed rats (Figures 6A,C). Although the same change tendency was observed in the CA2/3 and DG of the HIP, traumatic stress unchanged the number of intersections in the regions of CA2/3 (Ado: [*F*(2, 15) = 5.18, *P* > 0.05]; Adu: [*F*(2, 15) = 0.57, *P* > 0.05]) and DG (Ado: [*F*(2, 15) = 3.36, *P* > 0.05]; Adu: [*F*(2, 15) = 0.96, *P* > 0.05]) in both adolescent groups and adult groups (Supplementary Figure 1). In the PFC, traumatic stress also reduced the total number of intersections in adolescent rats [*F*(2, 15) = 6.40, *P* < 0.05] (*post hoc*, *P* = 0.0378) and Unc0642 administration mitigated these changes in adolescent rats (*post hoc*, *P* = 0.0113) compared with stressed rats. However, in the PFC of adult rats, traumatic stress reduced the total number of intersections [*F*(2, 15) = 13.62, *P* < 0.05] (*post hoc*, *P* = 0.0005) and the Unc0642 administration unimproved neuronal development (*post hoc*, *P* = 0.6008) compared with stressed rats (Figures 6B,D).

#### Traumatic Stress Procedure and Effect of Unc0642 on BDNF and BDNF mRNA Expression Both in Adolescent and Adult Rats

**FIGURE 7:**
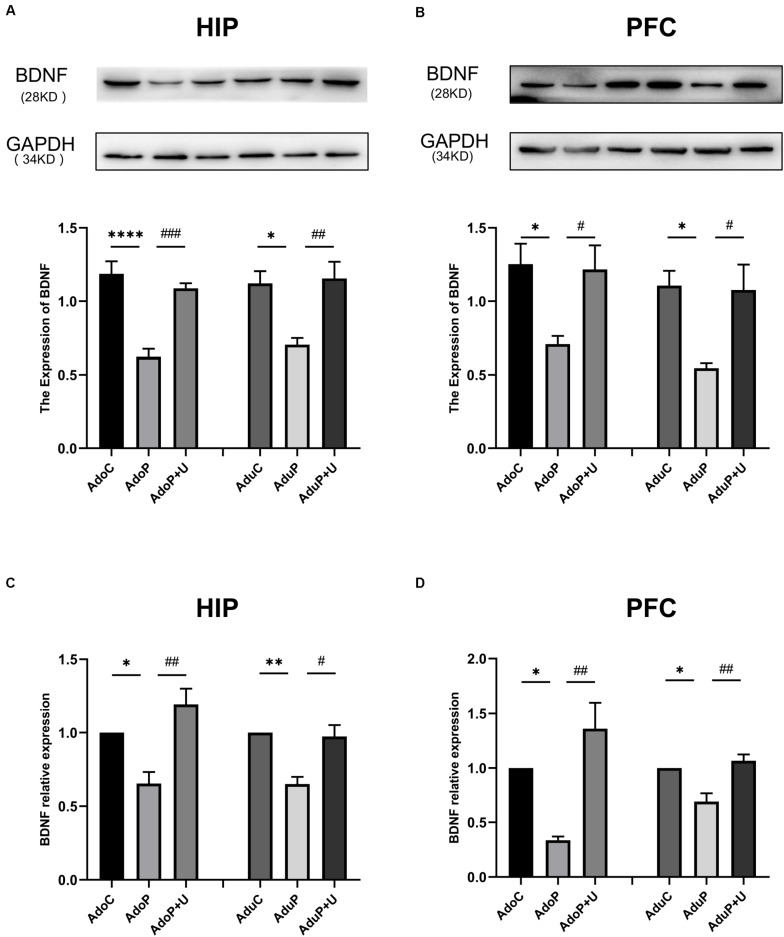
Traumatic stress procedure and effect of Unc0642 on BDNF protein and BDNF mRNA expression both in adolescent and adult rats. Results were presented as mean ± SEM (*n* = 4/3 each group). Levels of BDNF protein in the hippocampus **(A)** and PFC **(B)** were quantified and normalized to GAPDH. Levels of BDNF mRNA in the hippocampus **(C)** and PFC **(D)** were quantified and normalized to β-actin. *p < 0.05, **p < 0.01, ****p < 0.0001 and ^#^*p <* 0.05, ^##^*p <* 0.01, ^###^*p <* 0.001 (Tukey’s test).

The results of the analysis of the effects of IFS and treatment with Unc0642 on BDNF and BDNF mRNA expression are shown in Figure 7. In the HIP, traumatic stress decreased the expression of BDNF protein in adolescent rats [*F*(2, 12) = 24.03, *P* < 0.05] (*post hoc*, *P <* 0.0001) and treatment with Unc0642 relieved the change in adolescent rats (*post hoc*, *P* = 0.0005) compared with stressed rats. In adult groups, traumatic stress decreased the expression of BDNF protein [*F*(2, 12) = 8.39, *P* < 0.05] (*post hoc*, *P* = 0.0137) and treatment with Unc0642 mitigated this change in adult rats (*post hoc*, *P* = 0.0081) (Figure 7A). In the PFC, traumatic stress decreased the expression of BDNF protein in adolescent rats [*F*(2, 12) = 5.70, *P* < 0.05] (*post hoc*, *P* = 0.0268) and Unc0642 administration attenuated this change in adolescent rats (*post hoc*, *P* = 0.0038) compared with stressed rats. In the PFC of rats in adult groups, traumatic stress decreased the expression of BDNF protein [*F*(2, 12) = 7.08, *P* < 0.05] (*post hoc*, *P* = 0.0150) and treatment with Unc0642 alleviated this change in adult rats (*post hoc*, *P* = 0.0206) compared with stressed rats (Figure 7B). Additionally, in the HIP, traumatic stress decreased the expression of BDNF mRNA in adolescent rats [*F*(2, 6) = 12.74, *P* < 0.05] (*post hoc*, *P* = 0.0426) and Unc0642 administration relieved this change in adolescent rats (*post hoc*, *P* = 0.0060) compared with stressed rats. In the HIP of rats in the adult groups, traumatic stress also decreased the expression of BDNF mRNA [*F*(2, 6) = 13.65, *P* < 0.05] (*post hoc*, *P* = 0.0080) and treatment with Unc0642 mitigated this change in adult rats (*post hoc*, *P* = 0.0116) compared with stressed rats (Figure 7C). In the PFC, traumatic stress also decreased the expression of BDNF mRNA in adolescent rats [*F*(2, 6) = 14.33, *P* < 0.05] (*post hoc*, *P* = 0.0326) and treatment with Unc0642 mitigated this change in adolescent rats (*post hoc*, *P* = 0.0045) compared with stressed rats. In the PFC of rats in adult groups, traumatic stress decreased the expression of BDNF mRNA [*F*(2, 6) = 13.21, *P* < 0.05] (*post hoc*, *P* = 0.0174) and Unc0642 administration alleviated this change in adult rats (*post hoc*, *P* = 0.0071) (Figure 7D).

#### Traumatic Stress Procedure and Effect of Unc0642 on H3K9me2 Expression Both in Adolescent and Adult Rats

**FIGURE 8:**
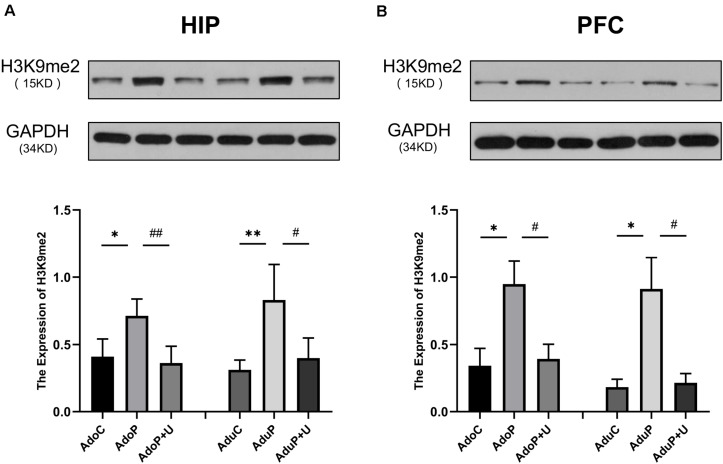
Traumatic stress procedure and effect of Unc0642 on H3K9me2 expression both in adolescent and adult rats. Results were presented as mean ± SEM (*n* = 4 each group). Levels of H3K9me2 protein in the hippocampus **(A)** and PFC **(B)** were quantified and normalized to GAPDH. *p < 0.05, **p < 0.01 and ^#^*p <* 0.05, ^##^*p <* 0.01 (Tukey’s test).

The results of the analysis of the effects of traumatic stress and treatment with Unc0642 on H3K9me2 expression are shown in Figure 8. In the HIP, traumatic stress increased the expression of H3K9me2 in adolescent rats [*F*(2, 9) = 8.88, *P* < 0.05] (*post hoc*, *P* = 0.0208) and treatment with Unc0642 mitigated this change (*post hoc*, *P* = 0.0093) compared with stressed rats. In the HIP of rats in adult groups, traumatic stress increased the expression of H3K9me2 [*F*(2, 9) = 9.516, *P* < 0.05] (*post hoc*, *P* = 0.0070) and Unc0642 administration relieved this change in adult rats (*post hoc*, *P* = 0.0199) compared with stressed rats (Figure 8A). In the PFC, traumatic stress increased the expression of H3K9me2 in adolescent rats [*F*(2, 9) = 5.87, *P* < 0.05] (*post hoc*, *P* = 0.0238) and Unc0642 administration attenuated this change in adolescent rats (*post hoc*, *P* = 0.0375) compared with stressed rats. In the PFC of rats in adult groups, traumatic stress increased the expression of H3K9me2 [*F*(2, 9) = 8.03, *P* < 0.05] (*post hoc*, *P* = 0.0155) and treatment of Unc0642 mitigated this change in adult rats (*post hoc*, *P* = 0.0197) (Figure 8B).

#### Traumatic Stress Procedure and Effect of Unc0642 on H3K9me2 Enrichment Both in Adolescent and Adult Rats

**FIGURE 9:**
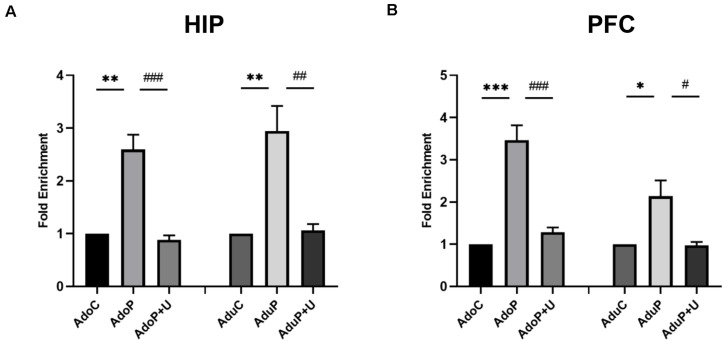
Traumatic stress procedure and effect of Unc0642 on H3k9me2 enrichment both in adolescent and adult rats. **(A)**. Fold enrichment of H3K9me2 detected by CHIP; **(B)**. Fold enrichment of H3K9me2 detected by CHIP. Results were presented as mean ± SEM (*n* = 3 each group). *p < 0.05, **p < 0.01, ***p < 0.001 and ^#^*p <* 0.05, ^##^*p <* 0.01, ^###^*p <* 0.001 (Tukey’s test).

The epigenetic marker H3K9me2 can bind to the promoter region of the *Bdnf* gene and reduce the BDNF mRNA and BDNF protein expression levels. After stress, the expression of H3K9me2 is increased and leads to decreased expression of BDNF. In the HIP, traumatic stress increased the enrichment of H3K9me2 in adolescent rats [*F*(2, 6) = 32.75, *P* < 0.05] (*post hoc*, *P* = 0.0013) and treatment with Unc0642 decreased the enrichment of H3K9me2 in adolescent rats (*post hoc*,

*P* = 0.0009) compared with stressed rats. In the HIP of rats in adult groups, traumatic stress increased the enrichment of H3K9me2 [*F*(2, 6) = 15.27, *P* < 0.05] (*post hoc*, *P* = 0.0067) and Unc0642 administration altered the enrichment in adult rats (*post hoc*, *P* = 0.0079) (Figure 9A). In the PFC, traumatic stress also increased the enrichment of H3K9me2 in adolescent rats [*F*(2, 6) = 40.0, *P* < 0.05] (*post hoc*, *P* = 0.0004) and treatment with Unc0642 decreased the enrichment of H3K9me2 in adolescent rats (*post hoc*, *P* = 0.0009) compared with stressed rats. Additionally, in the PFC of rats in adult groups, traumatic stress increased the enrichment of H3K9me2 [*F*(2, 6) = 9.023, *P* < 0.05] (*post hoc*, *P* = 0.0253) and Unc0642 administration altered this change in adult rats (*post hoc*, *P* = 0.0230) in PFC (Figure 9B). Therefore, this result directly confirmed that H3K9me2 regulates the expression level of *Bdnf* gene.

## Discussion

The present study found that adolescent traumatic stress induced anxiety, exploratory behavior suppression, reduction of social exploratory behavior and impairment of spatial memory in rats. Such findings indicate that IFS induced post-traumatic stress-like behavioral disorders in young rats, and the symptoms could persist into adulthood in rats (Wang R. et al., 2018). However, most of the above behaviors were improved by treatment with Unc0642, a small molecule inhibitor of EHMT2, which could reduce the protein expression level of H3K9me2. Together, these findings suggest that the H3K9me2/BDNF axis is involved in the regulation of the pathogenesis of PTSD.

Golgi staining is a well-known staining method that can clearly reveal the neuronal morphology. Golgi staining can identify neurons from axons, dendrites and other nerves interlaced around it (Das et al., 2013). In this study, the classic Sholl analysis of Golgi stained neurons was used to analyze the total number of intersections, dendrite length, and density of individual neurons in the HIP and PFC (Theer et al., 2014). The results showed that there are significant differences in the total number of intersections among groups, which is reflected in the length of each neuron and its degree of branch richness. IFS reduced the total number of intersections in the CA1 of HIP and PFC in adolescent and adult rats compared with control rats, and treatment with Unc0642 mitigated these changes in adolescent rats. However, the neuronal morphological changes in the HIP and PFC of adult rats were not alleviated by treatment with Unc0642. The result in adult rats was consistent with those of previous study showing that antidepressants do not reverse stress-induced neurogenesis disruption (Lino de Oliveira et al., 2020). Also, these results suggested that early life stress induces non reversible morphological changes in certain subregion of the brain which affect the susceptibility of an individual to PTSD. It is noticed that traumatic stress unchanged the number of intersections in the CA2/3 and DG of HIP in both adolescent and adult rats which is consisted with the conclusion that the HIP subfields specific response to stressors (Chen et al., 2018).

Previous studies have shown that lower BDNF levels in the brain are closely related to anxiety-like behaviors (Yu et al., 2012). Furthermore, decreased BDNF and TrkB levels induced by acute and chronic stress could result in dysregulation of neural development and neural plasticity, which are involved in the pathogenesis of affective disorders, such as depression (Luo et al., 2019). In the present study, PTSD reduced protein and mRNA expression levels of BDNF both in the HIP and PFC, which is consistent with similar findings in previous studies. Together the results of behavioral tests and morphology analysis suggest that the reduction of BDNF expression in the HIP and PFC induced by early traumatic stress is involved in the etiology and susceptibility to PTSD (Burstein et al., 2018; Lee et al., 2019). However, the change occurring at the epigenetic level requires further validation by experimental studies.

Dimethylated histone H3 lysine 9 (H3K9me2) is a critical epigenetic mark for gene repression and silencing (Chen et al., 2006). H3K9me2 is altered by oxidative stress and metal exposure (Chervona and Costa, 2012). Further, studies suggest that H3K9me2/3 is a regulator of the gene expressing BDNF (Tsankova et al., 2004; Zhou et al., 2017). Present study analyzed the H3K9me2 protein expression level by western blotting and H3K9me2 enrichment using CHIP. The results demonstrated that IFS in adolescence increased H3K9me2 protein level in both adolescent and adult stressed rats, and the higher H3K9me2 protein level was relieved after Unc0642 administration. The results indicated that traumatic stress experiences alter the epigenetic regulation of BDNF expression in the HIP and PFC. In addition, the CHIP analysis results showed both adolescent and adult rats in the stressed groups had higher levels of H3K9me2 enrichment, while Unc0642 administration reduced such increases. The results confirmed that H3K9me2 regulates the level of expression of the *Bdnf* gene. This means that the epigenetic marker H3K9me2 can directly silence the transcription of the *Bdnf* gene, thereby decreasing the expression of its mRNA and protein, which confirmed the mechanism of epigenetic regulation in acute trauma stress model.

In summary, our study demonstrates that IFS in adolescent male rats can induce post-traumatic stress-like behaviors and such symptoms can persist into adulthood. In addition, stressed rats displayed fewer intersections, the indicator of the length of the dendrites and branches of neuron in the CA1 of HIP and PFC region. Moreover, IFS also decreased the expression level of BDNF mRNA and protein, and increased the levels of H3K9me2. However, the small molecule inhibitor Unc0642 improved both behavioral and molecular biological indicators. The results indicate that the H3K9me2/BDNF axis is involved in dendritic development and synaptogenesis in the brain, which is related to pathogenesis of PTSD.

## Limitation

Present study has limitations. First, PTSD symptoms include depressive emotion (Wang W. et al., 2018; Richter-Levin et al., 2019), the sucrose consumption test and forced swimming test which can be used to evaluate the depression like behaviors of rats should be done. Second, previous studies show that the dorsal hippocampus has been implicated in memory function, while the ventral hippocampus has been implicated in stress and fear-related behaviors (Chen and Etkin, 2013; Chen et al., 2018). However, present study examined the expression of BDNF, BDNF mRNA and H3K9me2 enrichment in whole hippocampus, more precise study plan including injecting drug in the specific brain area should be done in the further. Finally, given our study only included male rats, the conclusion cannot be generalized to female population.

## Data Availability Statement

The raw data supporting the conclusions of this article will be made available by the authors, without undue reservation.

## Ethics Statement

The animal study was reviewed and approved by the Animal Ethics Committee of Shandong University.

## Author Contributions

FP was involved in the study design and data interpretation. MZ performed the majority of the laboratory work and contributed to the analysis of the data and writing the manuscript. WW, ZZ, ZJ, and DL were responsible for the animal model and behavioral tests. All authors contributed to the article and approved the submitted version.

## Conflict of Interest

The authors declare that the research was conducted in the absence of any commercial or financial relationships that could be construed as a potential conflict of interest.
