# Supplementary material for: Long-Term Effect of Post-traumatic Stress in Adolescence on Dendrite Development and H3K9me2/BDNF Expression in Male Rat Hippocampus and Prefrontal Cortex
Source: Front Cell Dev Biol. 2020 Jul 31;8:682. doi: 10.3389/fcell.2020.00682 (PMC7412801; doi:10.3389/fcell.2020.00682)
Supplement: Supplementary file 1 [file Data_Sheet_1.pdf]

Supplyment Figure 1.

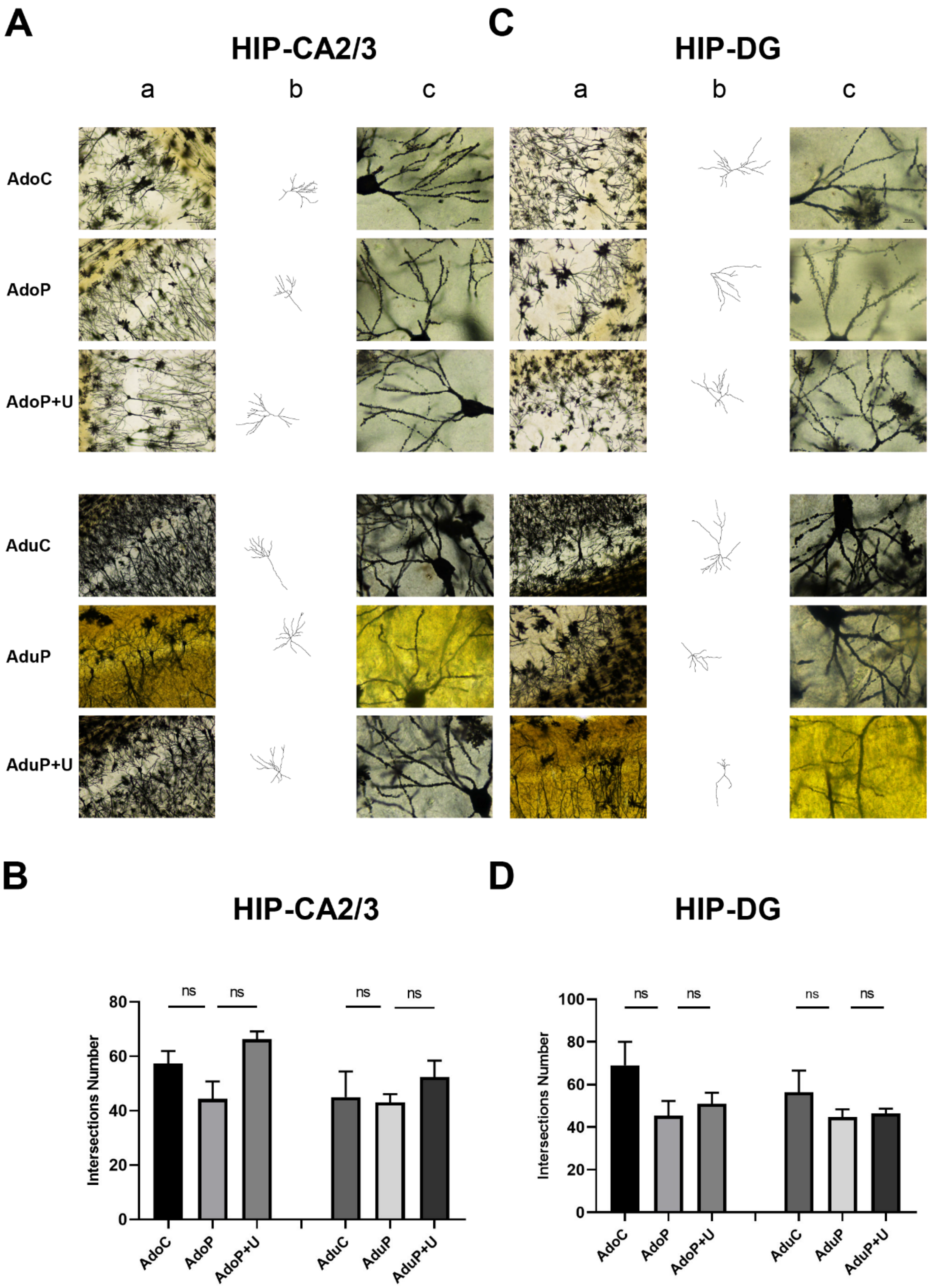

Supplementary Figure 1. Traumatic stress procedure and effect of Unc0642 on neuronal morphological changes of Golgi dyeing both in adolescent and adult rats. (A). Golgi dyeing in hippocampus-CA2/3 of adolescent and adult rats; (B). Intersections number of adolescent and adult rats; (C). Golgi dyeing in hippocampus-DG of adolescent and adult rats; (D). Intersections number of adolescent and adult rats. a. 200× in Golgi dyeing; b. Black and white picture; c. 1000× in Golgi dyeing. Results were presented as mean ± SEM (n = 6 each group) (ns: none significant).

Supplementary Figure 2.

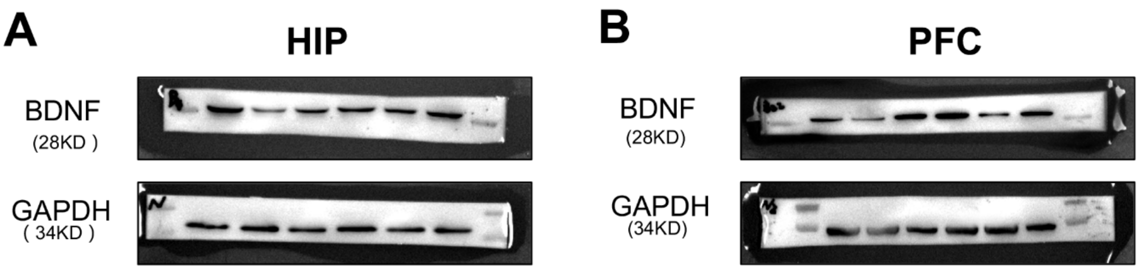

Supplementary Figure 2. Traumatic stress procedure and effect of Unc0642 on BDNF expression both in adolescent and adult rats. **(A).** BDNF expression in the hippocampus detected by western blotting. **(B).** BDNF expression in the prefrontal cortex detected by western blotting.

Supplementary Figure 3.

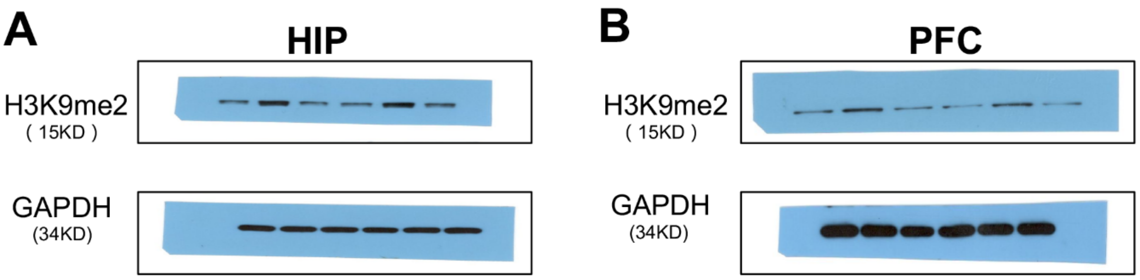

Supplementary Figure 3. Traumatic stress procedure and effect of Unc0642 on H3K9me2 expression both in adolescent and adult rats. **(A).** H3K9me2 expression in the hippocampus detected by western blotting. **(B).** H3K9me2 expression in the prefrontal cortex detected by western blotting.
